# False-positives and false-negatives in non-invasive prenatal testing (NIPT): what can we learn from a meta-analyses on > 750,000 tests?

**DOI:** 10.1186/s13039-022-00612-2

**Published:** 2022-08-19

**Authors:** Thomas Liehr

**Affiliations:** Institute of Human Genetics, Jena University Hospital, Friedrich Schiller University, Am Klinikum 1, 07747 Jena, Germany

**Keywords:** Non-invasive prenatal testing (NIPT), First trimester-screening (FTS), Teratogen effects, Multigenetic diseases, Pregnant woman perspective, False-positive, False-negative

## Abstract

**Background:**

Non-invasive prenatal testing (NIPT) has had an incomparable triumph in prenatal diagnostics in the last decade. Over 1400 research articles have been published, predominantly praising the advantages of this test.

**Methods:**

The present study identified among the 1400 papers 24 original and one review paper, which were suited to re-evaluate the efficacy of > 750,000 published NIPT-results. Special attention was given to false-positive and false-negative result-rates. Those were discussed under different aspects—mainly from a patient-perspective.

**Results:**

A 27: 1 rate of false-positive compared to false-negative NIPT results was found. Besides, according to all reported, real-positive, chromosomally aberrant NIPT cases, 90% of those would have been aborted spontaneously before birth. These findings are here discussed under aspects like (i) How efficient is NIPT compared to first trimester screening? (ii) What are the differences in expectations towards NIPT from specialists and the public? and (iii) There should also be children born suffering from not by NIPT tested chromosomal aberrations; why are those never reported in all available NIPT studies?

**Conclusions:**

Even though much research has been published on NIPT, unbiased figures concerning NIPT and first trimester screening efficacy are yet not available. While false positive rates of different NIPT tests maybe halfway accurate, reported false-negative rates are most likely too low. The latter is as NIPT-cases with negative results for tested conditions are yet not in detail followed up for cases with other genetic or teratogenic caused disorders. This promotes an image in public, that NIPT is suited to replace all invasive tests, and also to solve the problem of inborn errors in humans, if not now then in near future. Overall, it is worth discussing the usefulness of NIPT in practical clinical application. Particularly, asking for unbiased figures concerning the efficacy of first trimester-screening compared to NIPT, and for really comprehensive data on false-positive and false-negative NIPT results.

**Supplementary Information:**

The online version contains supplementary material available at 10.1186/s13039-022-00612-2.

## Introduction

The desire for prenatal information about the unborn child in the womb is probably as old as human history. While in ancient Greece, one could only consult an oracle or in the Middle Ages an astrologer, today's contemporaries have actually the opportunity to look into the belly of the expectant mother. This is possible for a little over half a century, still, meanwhile it is standard to obtain even cell material from the fetus and analyze it for its genetic integrity. Thus, for the first time in human history, largely reliable statements on the genetic health of an unborn child are possible [1]. The latter seemed unimaginable even for science fiction authors in the 1970s [2].

We are currently experiencing an increased worldwide demand for the earliest possible testing of unborn children [1]. This has various causes as:

(i) in industrialized countries many couples desire to have only one or at most two children, who then have to be healthy for sure;
(ii) there is increasing age of the first-time mothers, which at the same time demands minimizing an age-associated increased risk for the birth of a child with an aneuploidy;
(iii) in some so-called developing countries there is a desire of expectant parents to have male rather than female offspring and, if necessary, to be able to carry out abortion as early as possible; and/ or
(iv) nowadays apparently simple applicable, fast and new non-invasive prenatal diagnostic procedures (PNDPs) are available and widely used [1, 2].

At present, various invasive and non-invasive PNDPs are available. Standard invasive procedures include chorionic villus sampling, amniocentesis and umbilical cord blood sampling. In all three invasive methods, placental or fetal cells are examined cytogenetically, molecular cytogenetically, and/or molecular genetically. Only then, it is possible to make unambiguous statements on questions such as: Is there a trisomy, monosomy or a chromosomal rearrangement? Does the expectant child have a genetic deletion or duplication (smaller than an entire chromosome)? Is there a specific gene mutation? After successful completion of the corresponding invasive diagnostic procedures, unequivocal yes or no answers for the presence or absence of a genetic defect can be expected [1]. All non-invasive PNDPs, on the other hand, are so-called screening methods; thus, only a probability statement as to whether the child to be has a specific genetic change or not. These statements always need checking, ultimately through an invasive PNDP. The non-invasive PNDPs include all ultra-sonographic approaches, all biochemical tests from maternal blood (such as the determination of alpha-fetoprotein = AFP, beta-human chorionic gonadotropin = ß-hCG or pregnancy-associated plasma protein-A = PAPPA), all combined methods, such as triple test or first trimester screening (FTS), and also the latest instrument in this “kit”—non-invasive prenatal testing (NIPT). The latter belongs to non-invasive PNDPs because (i) not the genetic material of the expectant child itself but of circulating free DNA derived from the placenta is examined here, and (ii) no clear yes or no answers are obtained, as in invasive PNDPs, but only statements of probability [1–4].

**Fig. 1:**
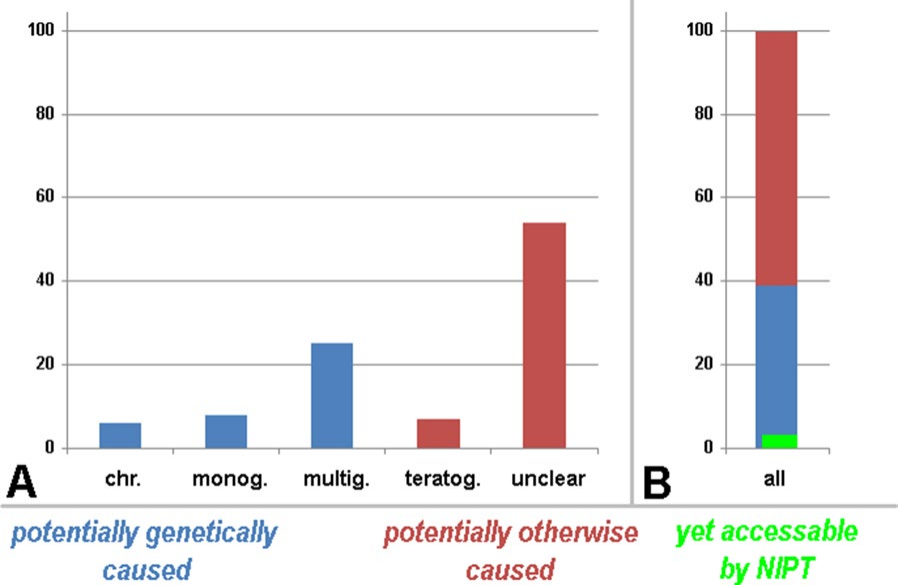
All newborns with clinical abnormalities and the causes of their problems are schematically depicted. In **A** the causes are specified as chromosomal aberrations (chr.), monogenetic (monog.), multigenetic (multig.), teratogenic (teratog.), and idiopathic (unclear) causes; in **B** all causes are summarized to 100%. It can be clearly seen that prenatally a maximum of 10% of the cases with clinical abnormalities can be characterized by a clear genetic cause; according to this review not more than 5% of these aberrant cases can be accessed by NIPT (green label)

Between 3 and 6% of newborns have major inborn abnormalities, which classifies them as “individuals with congenital diseases/disease complexes/syndromes” [4]. If one considers the 3–6% mentioned as an “own population”, then a chromosomal disorder is present in ~ 6%, a teratogenic damage in ~ 7%, and a monogenetic or multigenetic disease in ~ 8% or ~ 25%, respectively. For the remainder ~ 54%, the diagnosis usually stays lifelong as suffering from an “idiopathic disorder”, i.e. the cause remains unclear (Fig. 1). Prenatally accessible is rather a large part of the chromosomal disorders and a very small percentage of the monogenetic disease; in the best-case scenario, a clear prenatal genetic diagnosis can only be expected for up to 10% of newborns with birth defects [1]—see also Fig. 1.

In this context, the general statements, promises and enthusiasm on the impact of NIPT on prenatal diagnostics needs some investigation, especially as there is a big market interest and a huge amount of money involved here [5–7]. Thus, here the efficacy to identify prenatally individuals with congenital diseases is re-evaluated on > 750,000 published NIPT-results predominantly from China, Europe and USA. The reported false-positive (FP) and false-negative (FN) rates were summarized and are discussed here under several aspects. Overall, it seems that the usefulness of NIPT in practical clinical application needs to be fundamentally re-considered.

## Material and Methods

A Pubmed search for “non-invasive prenatal testing “identified slightly over 1400 publications in 05/2022; those were screened for studies reporting:

- more than 500 NIPT cases,
- on test results in (predominantly) singleton pregnancies,
- on NIPT-settings testing for trisomy 13 (T13), trisomy 18 (T18), trisomy 21 (T21), numerical sex-chromosome-aberrations (SCAs) and/or rare autosomal trisomies (RATs),
- on follow-up and/or an invasive PNDP to check for FP and FN cases in at least a subset of reported cases.

As listed in Additional file 1: Table S1 twenty-four corresponding studies were identified and included, originating from January 2017 to March 2022; also, a review (#25 in Additional file 1: Table S1) summarizing 32 corresponding studies before the year 2016 [8] was considered. Accordingly, > 750,000 NIPT cases are included and evaluated here.

## Results

The > 750,000 NIPT cases included in Additional file 1: Table S1, Tables. 1 and 2 were studied to different extend for (1) T13, (2) T18, (3) T21, (4) SCAs and/or (5) RATs. Thus, a separate evaluation was done for each of the five indications as well as for those three studies addressing all five indications simultaneously. For the latter group (Tables. 1a and 2a, Additional file 1: Table S1) of 41,472 cases tested by NIPT, 520 had a positive result. For 460/520 NIPT-positive cases (= 91.63%) a second, confirmatory test was done and 217 turned out to be FP (47.17%). Three cases were stated as having been attributed with a FN NIPT result (with respect to the NIPT-positive cases this would be 0.65%). To calculate a FP and FN rate corresponding to performed NIPT-cases only 91.63% of 41,472 = 38,001 cases were considered, as for the remainder cases no second confirmatory test has had been done (for different reasons). Accordingly, for all in parallel for T13, T18, T21, SCAs and RATs tested NIPT cases got in 1.211% a FP and 0.0079 a FN result.

**Table 1:** Based on a literature review (Additional file 1: Table S1) here numbers of NIPT-positive cases tested for the five indications trisomy 13, 18 and 21, SCAs and RATs are given together with false-positive and false-negative case-numbers

Indication	Tested overall	^+^91.63% of overall tested	Positive tested and checked	False positive	False negative
*a*					
all 5 indications tested	41,472	38,001	460^+^	217	3
*b*					
T13	721,157	639,378	687	304	13
T18	730,253	647,442	1447	268	30
T21	752,979	667,591	5283	517	31
SCAs	300,894	266,773	1192	661	1
RATs	158,395	140,433	342	289	0
all	n.a.	n.a.	8951*	2039	75

^*^All positive tested = 10,096 = 1.314%; ^+^checked positive NIPT cases: 88.66%

**Table 2:** Data from Table 1 is translated into percentages: false-positive and false-negative test results are expressed with respect to all cases tested by NIPT and with respect to all cases having a positive test result after NIPT

with respect to all NIPT tests Table 1 of corresp. tested cases [%]				with respect to Table 1 of corresp. tested cases [%]	
Indication	NIPT positives	False positive	False negative	False positive	False negative
*a*					
all	1.858	1.211	0.0079	47.17	0.65
*b*					
T13	0.101	0.048	0.0020	44.25	1.89
T18	0.228	0.041	0.0046	18.52	2.07
T21	0.796	0.077	0.0046	9.79	0.59
SCAs	0.448	0.248	0.0004	55.45	0.08
RATs	0.244	0.206	0.0000	84.50	0.00

The chromosome wise data for most common autosomal trisomies (Tables 1b and 2b, Additional file 1: Table S1) in ~ 720,000 to 752,000 cases tested by NIPT gave the following picture: 44.25%, 18.52% and 9.79% of NIPT results for T13, T18 and T21, respectively were FP. Concerning all NIPT positive cases 1.89%, 2.07% and 0.59%, each, were FN for the corresponding trisomies. Calculated for overall 88.66% cases tested by a second test if NIPT-positive, this equals to 0.048%, 0.041% and 0.077% FP and 0.020%, 0.046% and 0.046% for T13, T18 and T21, correspondingly.

For SCAs and RATs (Tables. 1b and 2b, Additional file 1: Table S1) the FN rates are low—only 1 case for SCAs is reported and none for RATs. However, between 55.45 or 84.50 women with a positive NIPT indeed learn after an invasive test that these results were FP for SCAs or RATs. Among all correspondingly tested women, this affects between 0.248 and 0.206% of them.

## Discussion

### General thoughts

When specialists trained in the field of human/ =clinical genetics discuss ethical considerations in prenatal diagnostics, at least one thing is common sense: It is always only about the actual pregnant woman or couple and the actual fetus/future child what is considered and has to be discussed [9]. It is not about costs and overall advantages of a test for society, or the financing of a health system. However, since introduction of NIPT, always clearly stated to be a screening and no diagnostic test, dozens of papers have been out discussing exactly these points in a patient- and individual-far way. There are studies on the identification of “the most relevant cost-effectiveness threshold” for T21 when using NIPT from France [10] or China [11, 12], or on the fact that “NIPT screening has a high health economical value “[13]. Against this argumentation, oppose Prinds et al. [14], who state that a counsellor can not be a healthcare information sharing communicator, but that the individual couple needs to be in the center of the counselling. At the same time, there are ongoing discussions on the negative influence of NIPT on the rights of people with disabilities [15, 16], on women’s autonomy [17] and on different ways how to integrate NIPT into a national ethical landscape [18].

Overall, all this is striking, and especially the numerous publications on the potential benefits of NIPT for the economy of the national health systems are somewhat surprising, as no similar movements were there when FTS was introduced, which could not be commercialized similarly, but had already high detection rates as a non invasive prenatal setting. The same way astonishing is that majority of NIPT related papers are extremely enthusiastic about the high sensitivity and specificity of NIPT for T21, but no papers ask why at the same time values for T13 and T18 are much less good, and for SCAs, RATs and microdeletion/microduplication syndromes are almost devastating. In addition, papers showing that the trophoblast, which shall be used for NIPT to characterize single gene mutations, has much higher and different mutation rates than cells of the same fetus, are not discussed in corresponding literature [19]. The here touched suspicious points on NIPT-application are discussed below based on the data from this meta-analyses.

*Conclusion*: The uncritical attitude of the majority of articles reporting NIPT introduction and utilization as a pure success story is at least surprising, if not conspicuous.

### Results of the present meta-analyses compared with literature data

Over 750,000 prenatal cases were reviewed here, to reevaluate the impact of NIPT on the detection of T13, T18, 21, SCAs and/or RATs. The possible differences in platforms and methods used for NIPT were intentionally not considered, even though they are well-known (to the author [20]). Such specification is normally also lacking in NIPT-related papers (see e.g. papers included as base for Additional file 1: Table S1). A more detailed, scientific unbiased comparison of capabilities of different NIPT-platforms would be critical, as in the literature it is rarely addressed [20] or done [21].

The two major findings of this meta-analysis are as follows:

- In this meta-analyses a 27: 1 rate of FP compared to FN NIPT result was found (Table 1 - 2,039: 75). Others [22], reviewing data under the aspect that real efforts to find FN cases were undertaken, found a higher 7.3: 1 rate of FP (88%) to FN (12%). However, Samura and Okamoto [23] report a comparable 30: 1 FP/FN rate concerning all tested women. In most studies it is not detailed how much effort was invested to find FN-cases; considering also the below treated question “Where are the remainder ‘abnormal newborns’ in the NIPT studies?” the data of Hartwig et al. [22] possibly may be closer to the true FN-rate than that of the present and the Samura and Okamoto [23] study.
- What in the present review was not considered are the also appearing 0.3-5.4% of NIPT cases which give no conclusive results (mainly due to underrepresentation of cffDNA (= cell free fetal DNA, as the placenta derived DNA is called in all literature,e in the sample), and the 0.1% of cases detecting maternal neoplasia or fetal chromosomal anomalies not intended to be checked [23]. Overall, one can expect to get for between 95 and 99% of the women tested with NIPT a negative result. In 1 to 5% the NIPT will be potentially positive and needs to be checked, which is done in the reviewed studies in ~ 90% of the cases.
- *Finding 1: According to the possibility of both FP and FN NIPT results, a positive as well as a negative NIPT-result with presence of sonographic abnormalities needs always to be checked by an invasive diagnostic.*
- According to Taylor-Phillips et al. [24] in the general obstetric population only can find in 100,000 pregnancies 40 cases with T13 (0.040%), 89 with T18 (0.089%) and 417 with T21 (0.417%). Nielsen and Wohlert [25] give a newborn frequency for SCAs of 0.0022%. Veropotvelyan and Nesterchuk [26] state for RATs 41% in missed abortions and in second trimester the percentage is 2.3%; data for newborns is not available. Moreover, the detection rate of numerical chromosomal aberrations in newborns overall is given as 0.6% [27]. As summarized in Table 3 NIPT for all numerical chromosomal aberrations detects 10.8 times more aberrant cases, than expected to be born. For T13, T18 and T21 two times as many than normally born children with these aneuploidies are picked up by NIPT, while for SCAs > 90 × more than abnormally born children are identified.
- *Finding 2: This data means that* > *90% of abnormal fetuses identified by NIPT would end up in abortion during the further pregnancy.*

**Table 3:** Data from Table 1 is translated into percentages for real positives among all tested cases compared to the expected rates acc. to the literature [24, 25, 27]

Indication	All NIPT positive with respect to Table 2 of corresp. tested cases [%]	Real positive with respect to Table 1 of corresp. tested cases [%]	Expected cases with respect to literature in newborns [%]	Difference for all NIPT positives [x more]	Difference for real positives [x more]
all (Tables 1a and 2)	1.858	0.6474	0.0600	31.0	10.8
T13	0.101	0.0619	0.0400	2.5	1.6
T18	0.228	0.1867	0.0890	2.6	2.1
T21	0.796	0.7186	0.4170	1.9	1.7
SCAs	0.448	0.1994	0.0022	203.0	90.6
RATs	0.244	0.0377	n.a	n.a	n.a

These findings and above-mentioned result lead to further questions as discussed now.

### What is the data meaning for pregnant women with a positive NIPT result?

Interestingly, this point is hardly treated in the literature, and especially the fact that only 10% of by NIPT detected aberrant fetuses would have a chance to be born is—to the best of the authors’ knowledge—not mentioned a single time in the 1400 papers screened for this review. As shown in Table 2, there are two ways to look at the FP and FN NIPT results; in most—if not all publications—NIPT is praised for 1.211% FP and 0.0079% FN cases. The fact that acc. to the tested questions almost 10% of women with a positive NIPT for T21 and almost 85% of them with a positive RAT-oriented NIPT were falsely alarmed that their baby carries a chromosomal abnormality—which is overall not less than ~ 2000 women among 750,000 tested ones—is normally not considered, addressed, or discussed. However, this is a complete perversion of the individual and patient-oriented view of clinical genetics. The latter is being engraved, as in most cases no adequate pre-NIPT counselling was offered, and also post-NIPT counselling often is not helpful for the pregnant woman. Women having had a false positive NIPT normally state they would never do the test again [20]. Furthermore, it must be suggested that in some settings, a positive NIPT is not tested by a second approach, meaning that indeed unaffected fetuses are aborted – see also the following section.

*Conclusion*: NIPT can only be offered together with detailed, qualified genetic counselling on possibilities, problems, shortcuts and consequences of the test in case of a positive test result; otherwise NIPT will be taken into account by pregnant women having wrong expectations.

### How much do pregnant women and their MDs trust the NIPT-result?

While performing a qualified NIPT includes always the mentioned qualified and extensive genetic counselling and an invasive test to confirm a positive result, it is at least doubtful if this guide is everywhere followed where NIPT is done. There are many reports of false positive NIPTs which were identified after the termination of a pregnancy (for review see [20], and also the Taiwanese study of Hsiao et al. [28] rather implies that pregnant women—guided by their MDs—rely on the NIPT completely, and tend to skip a confirmatory invasive test. Hsiao et al. [28] proudly presented that per 1 million women tested by double/ triple test with 1,051 suspected T21 cases 223 were born, while 1 million women tested with FTS identified 1680 suspected T21 cases with only 135 of them were born; this rate dropped in the next 1 million women with 1821 NIPT-positives for T21 to only 68 born T21 cases. For T18 the birth rates are given correspondingly as 19, 11, and 6 and for T13 as 8, 4 and 0. As the authors state, NIPT has a minimal false positive rate, and no own FP or FN data are provided in that report for their collective, these data at least raise the suspicion that invasive diagnostics was skipped in many of these cases and potentially healthy fetuses were terminated.

Besides this major trust of many MDs in NIPT results, the public and pregnant women are not well informed about the possibilities NIPT really comprises. This is only rarely systematically studied, but at least an influence of social class and education level on NIPT perception was shown in an US-study [29]. Another study done in Saudi Arabia showed that "the acceptance rate for NIPT is high, despite incomplete understanding of the benefits and limitations of the test" [30]; and similar results were obtained for pregnant women from China [31]. Bowman-Smart et al. [32] report for 34% of Australian women, interviewed after they gave birth to their child did not feel sufficiently informed of what the consequences of a positive NIPT result would have been.

*Conclusion*: Obstetricians and gynecologists need to be well-trained before offering NIPT to their patients. The public is relying on their expertise, which in this case is literally decisive over life or death. Also, MDs may get into juristic and financial troubles in case a FP or FN NIPT case becomes justiciable.

### What is the data meaning for pregnant women with a negative NIPT?

In NIPT-literature, this point is hardly covered. Still, Hirose et al. [33] showed that ~ 7% of Japanese women with a negative NIPT afterwards “regretted receiving NIPT and blamed themselves for taking it”, a result also found by Lo et al. [34]. This was mainly attributed to a lack of trustworthy genetic counselling and psychological support, which is necessary as pregnant women who undergo NIPT have greater stress and anxiety than pregnant women who do not [35]. Comparable studies for other countries besides Japan are not available, and acc. to Nakamura et al. [36] it has not been checked, if also in other cultural settings “more than one-third of the pregnant women who had a negative NIPT result still experienced strong state anxiety (transient anxiety in each period), even after disclosure of their results” [33]. Most other studies did not specifically ask about the feelings of the women after the test; however, in an US-study [37] also 30% of pregnant women reported “elevated anxiety at the time of testing”. Furthermore, an Australian study [32] found that “95% of respondents indicated they would undergo NIPT in a future pregnancy” while the remainder 5% had negative experiences with NIPT testing. It is not further discussed, if the 5% mainly included the FP, FN or other cases with non-informative NIPT results, and similar peculiarities discussed above.

Again, it is a single Japanese study, which dealt with reasons why women were unsettled by NIPT, Yotsumoto et al. [38] detail four major reasons as follows:

- lack of information from genetic counselling;
- feeling of social pressure not to have a child with a disability, especially T21;
- anxiety due to 2 weeks waiting for NIPT result coupled with an unawareness of doubts about the completeness of later obtained results;
- general doubts as " ‘options in the case of a positive result’, ‘guilt towards the child’, ‘criticisms on NIPT from others,’ ‘denial of disabled people’, and ‘how to tell the child’ “.

*Conclusion*: Obstetricians and gynecologists need NOT leave alone the women to whom they offered the test; at least one additional consultation appointment should be offered to the advisor after the NIPT was sent off and before the result is there.

### Where are the remainder ‘abnormal newborns’ in the NIPT studies?

In all here reviewed studies the pregnancies which showed no trisomies, SCAs, RATs (or other tested conditions not included in this review) were just reported as “normal after NIPT” or even “normal in follow-up”. As shown in Fig. 1 this is more than unlikely. Among 38,001 pregnancies (Table 1a) tested for all numerical whole chromosome aberrations with 37,755 NIPT negative cases there must be also children born suffering from microdeletion/ microduplication syndromes, monogenic and multigenetic disorders, as well as teratogenic and idiopathic conditions. If there are 246 cases found with real positive NIPT test—acc. to Table 3 about 25 of them would have been born acc. to data summarized in Taylor-Phillips et al. [24]. Among 38,001 born children, there must be an overall 1,140 to 2280 children with chromosomal aberrations, teratogenic damage, a monogenetic or multigenetic disease or an “idiopathic disorder” (Fig. 1).

*Conclusion*: NIPT studies must include all clinically abnormal cases—and if they group them all as idiopathic. Yet the published data is at least obviously incomplete, implying to the public a ‘wrong feeling of safety’.

### What about NIPT for microdeletion/microduplication syndrome detection and for monogenic disorders?

NIPT has a high FP rate for SCAs and RATs of 0.21% to 0.25% for all tested pregnancies and concerning those women getting a positive NIPT result, it is at 55.45 and 84.5, respectively (Table. 2). The rates are similar for microdeletion/microduplication syndrome detection. FP-rates of ~ 50% are reported for DiGeorge syndrome [39], and they get higher the rarer the tested syndrome is [40]. Scharf [4] states that such a high false positive rate of NIPT for this kind of indication means at the same time an unnecessary high invasive PNDP rate. Thus, German guidelines do recommend not to use of NIPT for screening of microdeletion-/microduplication syndromes [41].

NIPT for detection of single gene disorders and corresponding point mutations are most often referred to as NIPS, to distinguish from ‘normal NIPT’. While few authors report on a 100% detection rate for mutations in small proof of principle studies [42], others are more skeptical. Already in 2009 it was highlighted that NIPT latest then becomes complex when fetus and mother have the same alleles, which are indistinguishable on cffDNA level [43]. Recently, the confined placental mosaicism problem being responsible for major parts of FP-NIPT results was also treated for point mutations. Coorens et al. [19] showed that “every placental sample represents a clonal expansion that is genetically distinct and exhibits a genomic landscape akin to that of childhood cancer in terms of mutation burden and mutational imprints.” The authors state that tremendous mutagenesis is rather rule than exception in the placenta.

*Conclusion*: NIPT for microdeletion-/microduplication syndromes is leading to that many FP results that it rather increases invasive testing than reduces it; chromosome microarray based on invasively obtained fetal tissue would be most informative here. NIPT for single gene mutations is due to biological peculiarities of the placenta a priori prone to FP results due to clonal developments similar as in cancer tissue.

### Which data has a higher impact—NIPT or first trimester screening?

Conclusive data on the important question of which test is more reliable and more comprehensive, NIPT or FTS, are still lacking—there is only one mathematical algorithm based study [44] dealing with this problem. Kane et al. [44] suggest a 74% detection rate for T13, T18, T21 and 45,X for FTS compared to ~ 94% in NIPT. However, for other SCAs or RATs, FTS has a 35% detection rate compared to 0 or 13% in SNP- or non-SNP-based NIPT. Besides, Fries [45] showed in a small study on 153 cases picked up by FTS, that NIPT for T13, T18 and T21 would have missed at least 20% of those aberrant cases (like microdeletions or other chromosomal aberrations).

Nonetheless, while summarizing general prevalence thresholds of screening tests in obstetrics and gynecology Elfassy et al. [46] concluded that NIPT as it”is performed by sequencing fetal cell-free DNA in maternal circulation” (as we know this is not true) and “based on its inherent sensitivity and specificity parameters” (which are not supported by the present meta-analyses), NIPT to have a 2.4 times higher predictive value than FTS.

More realistically, Scharf [4] summarized that the detection rate of FTS for T21 is 95% and by NIPT 99%; thus he recommends T21-specific NIPT in combination with FTS, and especially with fetal sonography. Furthermore, Scharf [4] comes even to the conclusion that NIPT for T13 and T18 “no longer meet the quality criteria that are internationally applied to a screening procedure”.

Overall, two things are clear: (1) there was never a plea for FTS to finance by the health system like it was for NIPT, e.g. by Dutch MDs and scientists [47]; and (2) the best pickup-rate for chromosomal imbalances has the chromosome microarray combined with banding cytogenetics from amniocytes [48, 49].

*Conclusion*: An unbiased comparison of NIPT and FTS performance in the same patient population is necessary and still has not been published, even though data must be available.

### Why not invasive testing?

It has already repeatedly been stated (e.g. [20]) that actual invasive PNDP cannot be compared with that 30 or 50 years ago. As nicely summarized by Salomon et al. [50]: “the invasive procedure-related risk of fetal loss of 1%, which was a major argument to increase the use of NIPT, has been reviewed drastically down to around 1/1000 or less “(see [45]).

*Conclusion*: The risk of abortion is no longer an argument for NIPT and against invasive PNDP.

### Why are the false-positive rates so low for T21 and 2 to 6 times higher for all other aneuploidies?

All NIPT-related papers proudly present the almost perfect performance of the test for T21 with only an ~ 10% FP rate. The 2 to 9 times higher FP-rates for the remainder indications tested are only stated and hardly scientifically discussed. Some papers even neglect them and state that they found such reliable test systems and mathematical algorithms to come to 100% detection rates in NIPT for each indication and that they have no FP or FN results at all, which seems biologically impossible (keywords: confined placental mosaicism, vanishing twins, etc.) [51]. Most likely, especially the FP-data available from detailed NIPT results analyses could provide insights into trisomic rescue mechanisms in early embryogenesis. Eventually, even lesions could be learned which trisomies lead to fetal death first and which are more likely to be rescued, and why. Only one study states that 7 of 54 FP NIPT cases (13%) were due to vanishing twins [52].

*Conclusion*: NIPT was, is and will be a screening test. It detects placenta aberrations being present between the 11th and 25th weeks of gestation. All positive results must carefully be treated as possible HINT on an aberration in the fetus and a pointer that the early pregnancy might naturally end prematurely. Scientifically, the NIPT data has not been explored yet.

## Conclusion

Having discussed all the problems of NIPT, for individual settings and considering the ethical considerations under which each human genetic counsellor (MD or non-MD) is trained, it is really hard to understand that actual papers start with sentences like: “NIPT has revolutionized the approach to prenatal diagnosis and, to date, it is the most superior screening method for the common autosomal aneuploidies” [53]; or with: “our findings show the diversity of clinical practice with regard to the incorporation of NIPT into prenatal screening algorithms, and suggest that the use of NIPT both as a first-line screening tool in the general obstetric population, and to screen for SCAs and CNVs, is becoming increasingly prevalent” [54]. There is an urgent need to come back to the couple, patient and unborn child perspective and away from cost and profit argumentations. Finally, it is necessary to publish unbiased figures concerning (a) efficacy to identify potentially genetically aberrant fetuses when using FTS compared to NIPT, (b) FP-rates of different NIPT tests, (c) FN-rates of different NIPT tests including all test failures, and all cases with not tested chromosomal aberrations, and not tested monogenetic, multigenetic, teratogenic, and idiopathic (unclear) causes of inborn defects. Overall, the most likely intentionally induced impression in public that NIPT is or at least will be the PNDP performed in future must be revised quickly, to preserve the credibility of prenatal and clinical genetic diagnostics in the long term.

## Supplementary Information

**Additional file1: Table S1**. Here the data is summarized as extracted from 25 references (as listed below the table) on the results of NIPT. Absolute numbers are given here and no percentages. Abbreviations: FN = false negative; FP = false positive; n.a. = not available; n.d. = not determined; NIPT = non-invasive prenatal testing; RATs = rare autosomal trisomies (RATs); Ref = References; SCAs = numerical sex-chromosome-aberrations; T13 = trisomy 13; T18 = trisomy 18; T21 = trisomy 21.

## Data Availability

All data generated or analyzed during this study are included in this published article [and its supplementary information files].

## Abbreviations

AFP: Alpha-fetoprotein
ß-hCG: Beta-human chorionic gonadotropin
cffDNA: Cell free fetal DNA
DNA: Deoxyribonucleic acid
FN: False-negative
FP: False-positive
FTS: First trimester-screening
NIPT: Non-invasive prenatal testing
PAPPA: Pregnancy associated plasma protein-A
PNDP: Prenatal diagnostic procedure
RATs: Rare autosomal trisomies
SCAs: Numerical sex-chromosome-aberrations
T13: Trisomy 13
T18: Trisomy 18
T21: Trisomy 21
