# Supplementary material for: False-positives and false-negatives in non-invasive prenatal testing (NIPT): what can we learn from a meta-analyses on > 750,000 tests?
Source: Mol Cytogenet. 2022 Aug 19;15:36. doi: 10.1186/s13039-022-00612-2 (PMC9392255; doi:10.1186/s13039-022-00612-2)
Supplement: Supplementary file 1 — Additional file1: Table S1. Here the data is summarized as extracted from 25 references (as listed below the table) on the results of NIPT. Absolute numbers are given here and no percentages. Abbreviations: FN = false negative; FP = false positive; n.a. = not available; n.d. = not determined; NIPT = non-invasive prenatal testing; RATs = rare autosomal trisomies (RATs); Ref = References; SCAs = numerical sex-chromosome-aberrations; T13 = trisomy 13; T18 = trisomy 18; T21 = trisomy 21. [file 13039_2022_612_MOESM1_ESM.docx]

**Suppl. Tab. 1**

| **Ref.** | **Study performed in (Country)** | **T13** | | | **T18** | | | **T21** | | | **SCAs** | | | **RATs** | | | **NIPT cases** | **NIPT positive cases** | | **Positive NIPT cases checked by second test** | **Real positive of tested by second test** |
| --- | --- | --- | --- | --- | --- | --- | --- | --- | --- | --- | --- | --- | --- | --- | --- | --- | --- | --- | --- | --- | --- |
|  |  | **all** | **FP** | **FN** | **all** | **FP** | **FN** | **all** | **FP** | **FN** | **all** | **FP** | **FN** | **all** | **FP** | **FN** |  |  |  |  |  |
|  | China | 5 | 0 | 1 | 12 | 3 | 0 | 49 | 3 | 1 | 42 | 24 | 1 | 15 | 13 | 0 | 14,574 | 123 | 123 | | 77 |
|  | China | 34 | 24 | 0 | 43 | 15 | 2 | 150 | 28 | 0 | n.d. | n.d. | n.d. | n.d. | n.d. | n.d. | 40,613 | 257 | 227 | | 158 |
|  | China | n.d. | n.d. | n.d. | n.d. | n.d. | n.d. | n.d. | n.d. | n.d. | 193 | 126 | 0 | n.d. | n.d. | n.d. | 34,717 | 229 | 193 | | 67 |
|  | Italy | 27 | 5 | 0 | 62 | 6 | 0 | 247 | 2 | 0 | 117 | 18 | 0 | n.d. | n.d. | n.d. | 36,456 | 501 | 453 | | 422 |
|  | China | 3 | 2 | 0 | 13 | 5 | 0 | 32 | 5 | 0 | 78 | 48 | 0 | 34 | 16 | 0 | 17,428 | 202 | 160 | | 84 |
|  | China | 18 | 13 | 0 | 53 | 17 | 0 | 123 | 11 | 1 | n.d. | n.d. | n.d. | n.d. | n.d. | n.d. | 22,343 | 237 | 194 | | 152 |
|  | China | 26 | 4 | 0 | 34 | 3 | 0 | 40 | 1 | 0 | 28 | 3 | 0 | n.d. | n.d. | n.d. | 870 | 128 | 128 | | 117 |
|  | Japan | 55 | 26 | 0 | 37 | 18 | 1 | 717 | 9 | 2 | n.d. | n.d. | n.d. | n.d. | n.d. | n.d. | 43,133 | 809 | 809 | | 755 |
|  | China | 20 | 16 | 0 | 53 | 13 | 0 | 301 | 12 | 0 | n.d. | n.d. | n.d. | n.d. | n.d. | n.d. | 44.578 | 466 | 374 | | 333 |
|  | China | 28 | 24 | 0 | 27 | 14 | 0 | 125 | 20 | 0 | 218 | 142 | 0 | n.d. | n.d. | n.d. | 40,311 | 468 | 398 | | 198 |
|  | Italy | 3 | 1 | 0 | 7 | 2 | 0 | 62 | 4 | 0 | 13 | 5 | 0 | n.d. | n.d. | n.d. | 7,108 | 85 | 85 | | 73 |
|  | China | 21 | 18 | 0 | 29 | 10 | 0 | 74 | 14 | 0 | 137 | 57 | 0 | n.d. | n.d. | n.d. | 42,894 | 261 | 261 | | 162 |
|  | China | 14 | 8 | 0 | 18 | 7 | 0 | 55 | 9 | 0 | 34 | 23 | 0 | n.d. | n.d. | n.d. | 14,047 | 148 | 121 | | 74 |
|  | China | 24 | 4 | 0 | 37 | 21 | 0 | 121 | 99 | 0 | n.d. | n.d. | n.d. | 105 | 95 | 0 | 774 | 287 | 287 | | 65 |
|  | Korea | 3 | 1 | 0 | 8 | 0 | 1 | 21 | 1 | 0 | n.d. | n.d. | n.d. | n.d. | n.d. | n.d. | 1,011 | 32 | 32 | | 30 |
|  | China | 29 | 25 | 0 | 31 | 14 | 0 | 130 | 27 | 0 | 112 | 75 | 0 | 32 | 29 | 0 | 42,910 | 534 | 334 | | 164 |
|  | Netherlands | 55 | 24 | 0 | 49 | 1 | 5 | 239 | 9 | 5 | n.d. | n.d. | n.d. | 101 | 91 | 0 | 73,239 | 444 | 444 | | 135 |
|  | China | 17 | 7 | 0 | 11 | 2 | 0 | 50 | 1 | 0 | 44 | 43 | 0 | 55 | 45 | 0 | 9,470 | 177 | 177 | | 98 |
|  | USA | 31 | 16 | 2 | 53 | 8 | 5 | 153 | 7 | 3 | n.d. | n.d. | n.d. | n.d. | n.d. | n.d. | 58,048 | 572 | 237 | | 206 |
|  | China | 15 | 8 | 0 | 36 | 11 | 0 | 113 | 18 | 1 | 143 | 82 | 0 | n.d. | n.d. | n.d. | 31,515 | 434 | 307 | | 188 |
|  | Korea | 9 | 1 | 0 | 42 | 0 | 0 | 57 | 1 | 0 | n.d. | n.d. | n.d. | n.d. | n.d. | n.d. | 1,055 | 108 | 108 | | 106 |
|  | China | 5 | 4 | 0 | 9 | 3 | 0 | 30 | 0 | 1 | 33 | 15 | 0 | n.d. | n.d. | n.d. | 8,594 | 88 | 77 | | 55 |
|  | Japan | 44 | 16 | 0 | 128 | 22 | 1 | 289 | 10 | 1 | n.d. | n.d. | n.d. | n.d. | n.d. | n.d. | 30,613 | 545 | 461 | | 413 |
|  | China | 1 | 1 | 0 | 4 | 3 | 0 | 12 | 0 | 0 | n.d. | n.d. | n.d. | n.d. | n.d. | n.d. | 1,874 | 17 | 17 | | 13 |
| 1. * | Europe/ USA/ China | 200 | 56 | 10 | n.d. | n.d. | n.d. | n.d. | n.d. | n.d. | n.d. | n.d. | n.d. | n.d. | n.d. | n.d. | 137,699** | 200 | 200 | | 134 |
|  |  | n.d. | n.d. | n.d. | 651 | 70 | 15 | n.d. | n.d. | n.d. | n.d. | n.d. | n.d. | n.d. | n.d. | n.d. | 146,795** | 651 | 651 | | 566 |
|  |  | n.d. | n.d. | n.d. | n.d. | n.d. | n.d. | 2,093 | 226 | 16 | n.d. | n.d. | n.d. | n.d. | n.d. | n.d. | 169,521** | 2,093 | 2,093 | | 1,839 |
| **Overall** |  | **687** | **304** | **13** | **1,447** | **268** | **30** | **5,283** | **517** | **31** | **1,192** | **661** | **1** | **342** | **289** | **0** | **768,175** | **11,043** | **8,951** | | **6,684** |

* meta-analysis; ** partially overlapping figures – summarized as overall 150,000 cases

References

1. Chen Y, Yang F, Shang X, Liu S, Li M, Zhong M. A study on non-invasive prenatal screening for the detection of aneuploidy. Ginekol Pol. 2022 Mar 22. doi: 10.5603/GP.a2021.0254. Epub ahead of print. PMID: 35315016.
2. Wang D, Yang J, Peng H, Hou Y, Wang Y. [Analysis of the results of chromosomal trisomies 21, 18 and 13 screening among 40 628 women by non-invasive prenatal testing]. Zhonghua Yi Xue Yi Chuan Xue Za Zhi. 2021 Nov 10;38(11):1045-1050. Chinese. doi: 10.3760/cma.j.cn511374-20200819-00610. PMID: 34729740.
3. Luo Y, Hu H, Zhang R, Ma Y, Pan Y, Long Y, Hu B, Yao H, Liang Z. An assessment of the analytical performance of non-invasive prenatal testing (NIPT) in detecting sex chromosome aneuploidies: 34,717-patient sample in a single prenatal diagnosis Centre in China. J Gene Med. 2021 Sep;23(9):e3362. doi: 10.1002/jgm.3362. Epub 2021 Jun 14. PMID: 33973298.
4. La Verde M, De Falco L, Torella A, Savarese G, Savarese P, Ruggiero R, Conte A, Fico V, Torella M, Fico A. Performance of cell-free DNA sequencing-based non-invasive prenatal testing: experience on 36,456 singleton and multiple pregnancies. BMC Med Genomics. 2021 Mar 30;14(1):93. doi: 10.1186/s12920-021-00941-y. PMID: 33785045; PMCID: PMC8011149.
5. Dai R, Yu Y, Zhang H, Li L, Jiang Y, Liu R, Zhang H. Analysis of 17,428 pregnant women undergoing non-invasive prenatal testing for fetal chromosome in Northeast China. Medicine (Baltimore). 2021 Feb 12;100(6):e24740. doi: 10.1097/MD.0000000000024740. PMID: 33578623.
6. Zhu H, Jin X, Xu Y, Zhang W, Liu X, Jin J, Qian Y, Dong M. Efficiency of non-invasive prenatal screening in pregnant women at advanced maternal age. BMC Pregnancy Childbirth. 2021 Jan 26;21(1):86. doi: 10.1186/s12884-021-03570-6. PMID: 33499806; PMCID: PMC7836475.
7. Bu J, Jiang P, Cui X, Zhou H, Han F. Application values of prenatal screening and non-invasive gene sequencing in fetal birth defects. Pak J Med Sci. 2020 Nov-Dec;36(7):1545-1549. doi: 10.12669/pjms.36.7.2290. PMID: 33235572; PMCID: PMC7674863.
8. Suzumori N, Sekizawa A, Takeda E, Samura O, Sasaki A, Akaishi R, Wada S, Hamanoue H, Hirahara F, Sawai H, Nakamura H, Yamada T, Miura K, Masuzaki H, Nakayama S, Kamei Y, Namba A, Murotsuki J, Yamaguchi M, Tairaku S, Maeda K, Kaji T, Okamoto Y, Endo M, Ogawa M, Kasai Y, Ichizuka K, Yamada N, Ida A, Miharu N, Kawaguchi S, Hasuo Y, Okazaki T, Ichikawa M, Izumi S, Kuno N, Yotsumoto J, Nishiyama M, Shirato N, Hirose T, Sago H. Retrospective details of false-positive and false-negative results in non-invasive prenatal testing for fetal trisomies 21, 18 and 13. Eur J Obstet Gynecol Reprod Biol. 2021 Jan;256:75-81. doi: 10.1016/j.ejogrb.2020.10.050. Epub 2020 Oct 27. PMID: 33171421.
9. Xu H. [Retrospective analysis of 44 578 pregnancies undergoing non-invasive prenatal testing in Weifang]. Zhonghua Yi Xue Yi Chuan Xue Za Zhi. 2020 Oct 10;37(10):1065-1068. Chinese. doi: 10.3760/cma.j.cn511374-20191230-00672. PMID: 32924102.
10. Luo Y, Hu H, Jiang L, Ma Y, Zhang R, Xu J, Pan Y, Long Y, Yao H, Liang Z. A retrospective analysis the clinic data and follow-up of non-invasive prenatal test in detection of fetal chromosomal aneuploidy in more than 40,000 cases in a single prenatal diagnosis center. Eur J Med Genet. 2020 Sep;63(9):104001. doi: 10.1016/j.ejmg.2020.104001. Epub 2020 Jul 2. PMID: 32622960.
11. Mesoraca A, Margiotti K, Dello Russo C, Cesta A, Cima A, Longo SA, Barone MA, Viola A, Sparacino D, Giorlandino C. Cell-free DNA screening for aneuploidies in 7113 pregnancies: single Italian centre study. Genet Res (Camb). 2020 Jun 16;102:e5. doi: 10.1017/S001667232000004X. PMID: 32539871; PMCID: PMC7303798.
12. Liu Y, Liu H, He Y, Xu W, Ma Q, He Y, Lei W, Chen G, He Z, Huang J, Liu J, Liu Y, Huang Q, Yu F. Clinical performance of non-invasive prenatal served as a first-tier screening test for trisomy 21, 18, 13 and sex chromosome aneuploidy in a pilot city in China. Hum Genomics. 2020 Jun 5;14(1):21. doi: 10.1186/s40246-020-00268-2. PMID: 32503639; PMCID: PMC7275506.
13. Lu Z, Tian L, Ying H, Huang F. [Analysis of non-invasive prenatal testing in 14 047 cases of advanced age pregnant women]. Zhonghua Yi Xue Yi Chuan Xue Za Zhi. 2020 Jun 10;37(6):613-616. Chinese. doi: 10.3760/cma.j.issn.1003-9406.2020.06.004. PMID: 32472535.
14. Zhu X, Chen M, Wang H, Guo Y, Chau MHK, Yan H, Cao Y, Kwok YKY, Chen J, Hui ASY, Zhang R, Meng Z, Zhu Y, Leung TY, Xiong L, Kong X, Choy KW. Clinical utility of expanded non-invasive prenatal screening and chromosomal microarray analysis in high-risk pregnancy. Ultrasound Obstet Gynecol. 2021 Mar;57(3):459-465. doi: 10.1002/uog.22021. PMID: 32198896.
15. Hu HJ, Lee MY, Cho DY, Oh M, Kwon YJ, Han YJ, Ryu HM, Kim YN, Won HS. Prospective clinical evaluation of Momguard non-invasive prenatal test in 1011 Korean high-risk pregnant women. J Obstet Gynaecol. 2020 Nov;40(8):1090-1095. doi: 10.1080/01443615.2019.1680617. Epub 2019 Dec 12. PMID: 31826681.
16. Chen Y, Yu Q, Mao X, Lei W, He M, Lu W. Noninvasive prenatal testing for chromosome aneuploidies and subchromosomal microdeletions/microduplications in a cohort of 42,910 single pregnancies with different clinical features. Hum Genomics. 2019 Nov 29;13(1):60. doi: 10.1186/s40246-019-0250-2. PMID: 31783780; PMCID: PMC6884830.
17. van der Meij KRM, Sistermans EA, Macville MVE, Stevens SJC, Bax CJ, Bekker MN, Bilardo CM, Boon EMJ, Boter M, Diderich KEM, de Die-Smulders CEM, Duin LK, Faas BHW, Feenstra I, Haak MC, Hoffer MJV, den Hollander NS, Hollink IHIM, Jehee FS, Knapen MFCM, Kooper AJA, van Langen IM, Lichtenbelt KD, Linskens IH, van Maarle MC, Oepkes D, Pieters MJ, Schuring-Blom GH, Sikkel E, Sikkema-Raddatz B, Smeets DFCM, Srebniak MI, Suijkerbuijk RF, Tan-Sindhunata GM, van der Ven AJEM, van Zelderen-Bhola SL, Henneman L, Galjaard RH, Van Opstal D, Weiss MM; Dutch NIPT Consortium. TRIDENT-2: National Implementation of Genome-wide Non-invasive Prenatal Testing as a First-Tier Screening Test in the Netherlands. Am J Hum Genet. 2019 Dec 5;105(6):1091-1101. doi: 10.1016/j.ajhg.2019.10.005. Epub 2019 Nov 7. PMID: 31708118; PMCID: PMC6904791.
18. Zhou Y, Wang Z, Mao Q, Shi D, Zhang L, Xu L, Li H. [The value of non-invasive prenatal testing for the identification of fetal chromosome aneuploidies]. Zhonghua Yi Xue Yi Chuan Xue Za Zhi. 2019 Nov 10;36(11):1094-1096. Chinese. doi: 10.3760/cma.j.issn.1003-9406.2019.11.009. PMID: 31703133.
19. Hancock S, Ben-Shachar R, Adusei C, Oyolu CB, Evans EA, Kang HP, Haverty C, Muzzey D. Clinical experience across the fetal-fraction spectrum of a non-invasive prenatal screening approach with low test-failure rate. Ultrasound Obstet Gynecol. 2020 Sep;56(3):422-430. doi: 10.1002/uog.21904. PMID: 31671482; PMCID: PMC7496885.
20. Xu L, Huang H, Lin N, Wang Y, He D, Zhang M, Chen M, Chen L, Lin Y. Non-invasive cell-free fetal DNA testing for aneuploidy: multicenter study of 31 515 singleton pregnancies in southeastern China. Ultrasound Obstet Gynecol. 2020 Feb;55(2):242-247. doi: 10.1002/uog.20416. Epub 2020 Jan 8. PMID: 31364782.
21. Lee DE, Kim H, Park J, Yun T, Park DY, Kim M, Ryu HM. Clinical Validation of Non-Invasive Prenatal Testing for Fetal Common Aneuploidies in 1,055 Korean Pregnant Women: a Single Center Experience. J Korean Med Sci. 2019 Jun 24;34(24):e172. doi: 10.3346/jkms.2019.34.e172. PMID: 31222985; PMCID: PMC6589404.
22. Zheng Y, Wan S, Dang Y, Song T, Chen B, Zhang J. Non-invasive prenatal testing for detection of trisomy 13, 18, 21 and sex chromosome aneuploidies in 8594 cases. Ginekol Pol. 2019;90(5):270-273. doi: 10.5603/GP.2019.0050. PMID: 31165466.
23. Samura O, Sekizawa A, Suzumori N, Sasaki A, Wada S, Hamanoue H, Hirahara F, Sawai H, Nakamura H, Yamada T, Miura K, Masuzaki H, Nakayama S, Okai T, Kamei Y, Namba A, Murotsuki J, Tanemoto T, Fukushima A, Haino K, Tairaku S, Matsubara K, Maeda K, Kaji T, Ogawa M, Osada H, Nishizawa H, Okamoto Y, Kanagawa T, Kakigano A, Kitagawa M, Ogawa M, Izumi S, Katagiri Y, Takeshita N, Kasai Y, Naruse K, Neki R, Masuyama H, Hyodo M, Kawano Y, Ohba T, Ichizuka K, Kido Y, Fukao T, Miharu N, Nagamatsu T, Watanabe A, Hamajima N, Hirose M, Sanui A, Shirato N, Yotsumoto J, Nishiyama M, Hirose T, Sago H. Current status of non-invasive prenatal testing in Japan. J Obstet Gynaecol Res. 2017 Aug;43(8):1245-1255. doi: 10.1111/jog.13373. Epub 2017 Jun 6. PMID: 28586143.
24. Qiang R, Cai N, Wang X, Wang L, Cui K, Wang W, Wang X, Li X. Detection of trisomies 13, 18 and 21 using non-invasive prenatal testing. Exp Ther Med. 2017 May;13(5):2304-2310. doi: 10.3892/etm.2017.4272. Epub 2017 Mar 28. PMID: 28565842; PMCID: PMC5443185.
25. Iwarsson E, Jacobsson B, Dagerhamn J, Davidson T, Bernabé E, Heibert Arnlind M. Analysis of cell-free fetal DNA in maternal blood for detection of trisomy 21, 18 and 13 in a general pregnant population and in a high risk population - a systematic review and meta-analysis. Acta Obstet Gynecol Scand. 2017 Jan;96(1):7-18. doi: 10.1111/aogs.13047. Epub 2016 Dec 9. PMID: 27779757.
